# Development and validation of a risk stratification model for mild cognitive impairment among older adults with possible sarcopenia integrating traditional Chinese medicine constitution: a multicenter cross-sectional study

**DOI:** 10.3389/fpubh.2026.1980675

**Published:** 2026-10-30

**Authors:** Ruoli Wang, Nanping Mao, Yanyun He, Yi Wang, Wenbin Wu

**Affiliations:** 1 School of Clinical Medicine, Chengdu University of Traditional Chinese Medicine, Chengdu, China; 2 The Affiliated Hospital of Chengdu University of Traditional Chinese Medicine, Chengdu, China

**Keywords:** machine learning, mild cognitive impairment, possible sarcopenia, risk stratification, traditional Chinese medicine constitution

## Abstract

**Background:**

Possible sarcopenia is associated with cognitive impairment among older adults, while mild cognitive impairment (MCI) represents an important window for early identification. Existing MCI assessment approaches may depend on neuroimaging, genetic testing, or biochemical biomarkers, limiting their use in large-scale screening. Traditional Chinese medicine (TCM) constitution may provide accessible information on individual physiological and functional characteristics, but it has rarely been incorporated into MCI risk stratification.

**Objective:**

To develop and validate a machine learning-based risk stratification model integrating TCM constitution and conventional health-related indicators for MCI among older adults with possible sarcopenia.

**Methods:**

This multicenter cross-sectional study included 3,310 older adults with possible sarcopenia from ten tertiary hospitals in China and an independent single-institution external validation sample of 314 participants. The concurrent outcome was screening-defined probable MCI based on a prespecified Montreal Cognitive Assessment (MoCA) criterion. After a stratified 7:3 training–internal-validation split, data-dependent preprocessing parameters and least absolute shrinkage and selection operator (LASSO) feature selection were estimated using the training data only. Multilayer perceptron (MLP), gradient boosting decision tree (GBDT), support vector machine, K-nearest neighbors, logistic regression, and AdaBoost models were fitted using prespecified settings and evaluated using discrimination, classification, calibration, and decision-curve measures. SHapley Additive exPlanations (SHAP) were used only to describe contributions to model output.

**Results:**

Seven features were retained: educational attainment, sex, smartphone usage, Pittsburgh Sleep Quality Index score, Patient Health Questionnaire-9 score, qi-deficiency constitution, and damp-heat constitution. The MLP showed the highest internal discrimination, with an AUC of 0.853 (95% CI: 0.827–0.878); its AUC was 0.799 (95% CI: 0.773–0.823) in the external sample. The internal and external Brier scores were 0.168 and 0.151, calibration intercepts were −0.07 and 0.18, and calibration slopes were 0.80 and 1.07, respectively. SHAP analysis ranked feature contributions to model output and was not interpreted causally.

**Conclusion:**

This cross-sectional screening framework integrated TCM constitution with conventional indicators to stratify the probability of current screening-defined probable MCI among older adults with possible sarcopenia. The single-institution external evaluation supports only limited transportability; prospective multicenter evaluation and, where needed, recalibration are required before clinical implementation.

## 1 Introduction

Mild cognitive impairment (MCI) is recognized as an important intermediate stage between normal cognitive aging and dementia, although its clinical course is heterogeneous (1). Sociodemographic characteristics have been associated with incident MCI across several cohorts of older Chinese adults, highlighting the relevance of accessible population-level cognitive-status stratification (2). The American Academy of Neurology guideline reported that 14.4%–55.6% of individuals with MCI reverted to normal cognition during follow-up, while noting that some remained at increased risk of subsequent cognitive decline (3). Non-pharmacological management commonly includes physical exercise and cognitive training, and combined physical–cognitive interventions have been evaluated in older adults with MCI (4). The Finnish Geriatric Intervention Study to Prevent Cognitive Impairment and Disability (FINGER) also demonstrated cognitive benefits from an integrated program of nutritional guidance, physical activity, cognitive training, and vascular risk monitoring (5). Pharmacological disease-modification strategies remain an active area of investigation (6). Together, these findings support the importance of accessible approaches for identifying older adults who are more likely to have concurrent cognitive impairment.

Existing approaches for MCI-related stratification can be broadly classified into five categories: (1) demographic-only models; (2) cognition-based models incorporating cognitive test scores with or without subjective memory complaints or demographic data; (3) health variable models utilizing self-reported or objectively measured health status; (4) genetic-information models incorporating APOE, PICALM, and other Alzheimer's disease-associated genes (BIN1, CR1, ABCA7, MS4A6A, MS4A4E, CD33), either alone or in combination with non-genetic variables; and (5) multivariable models integrating demographic, health, and lifestyle measures (7).

However, some MCI stratification approaches incorporate information such as MRI-derived brain measures and genetic characteristics, as illustrated by large population cohorts including the Rotterdam Study (8). Research frameworks for the biological characterization of Alzheimer disease also emphasize cerebrospinal-fluid and imaging biomarkers (9). Although such measures are scientifically informative, their equipment, procedural, and technical requirements may limit their use in large-scale community screening. In addition, models developed in one population may not transfer directly to populations with different demographic, cultural, and health characteristics. Existing MCI risk-stratification models have rarely incorporated TCM constitution as an accessible population-level phenotype.

Previous studies have reported cross-sectional associations between TCM constitutional characteristics and cognitive status. Deng et al. found that yang-deficiency constitution, phlegm-dampness constitution, and blood-stasis constitution, together with elevated homocysteine, were associated with MCI status (10). Wang et al. described the development of the nine-category TCM constitution framework and its application to the study of individual physiological and psychological characteristics (11). More recent research has also reported associations between TCM constitution, mental health, and sleep quality (12). This evidence supports further evaluation of TCM constitutional assessment as an accessible individualized health indicator for cognitive risk stratification, without establishing a causal relationship with MCI.

A longitudinal study reported an MCI incidence of 24.2% among participants with sarcopenia (13), supporting an epidemiological association between the two conditions. Proposed pathways linking sarcopenia with cognitive impairment include chronic inflammation, mitochondrial dysfunction, oxidative stress, and altered muscle–brain signaling (14). These mechanisms remain biologically plausible explanations rather than causal pathways established by the present cross-sectional study.

Our model is designed to identify this priority screening population by integrating multiple dimensions of assessment, including clinical evaluations (such as sleep quality and depression symptom scores) and social determinants (such as educational attainment and digital device usage). Furthermore, it incorporates TCM constitutional characteristics as an individualized and accessible health indicator based on previous evidence.

The research highlights are reflected in three key aspects: (1) identifying older adults with possible sarcopenia as a priority group for MCI screening; (2) quantifying individual cognitive vulnerability through the lens of TCM constitutional theory; and (3) evaluating model transportability through multicenter data and independent external validation.

## 2 Methods

### 2.1 Data sources

This multicenter cross-sectional study enrolled 3,310 older adults (mean age 70.6 ± 7.7 years) meeting possible sarcopenia criteria per the AWGS 2019 diagnostic thresholds (grip strength <28 kg for males, <18 kg for females) (15). Participants were recruited from ten tertiary hospitals specialized in geriatric care and integrative medicine between January 2021 and December 2023. Each participant underwent a single study assessment; therefore, the variables and cognitive outcome represent approximately concurrent measurements rather than a longitudinal sequence. Participating institutions included academic medical centers located across China (complete list provided in Supplementary Table S1). The study protocol received ethical approval from the Institutional Review Board at Chengdu University of Traditional Chinese Medicine Affiliated Hospital (No. 2021KL-055) in compliance with the Declaration of Helsinki. Assessments included grip strength measured with a Xiangshan^®^ mechanical hand dynamometer, MoCA screening (16, 17), and TCM constitution assessment using Wang Qi's Body Constitution Classification Questionnaire (Supplementary Appendix S1).

### 2.2 Study eligibility criteria

This study employed the Xiangshan^®^ mechanical handgrip dynamometer to measure grip strength, with participants maintaining a seated position and elbow joint flexion of 90°. The maximum value from three consecutive trials of the dominant hand was recorded. According to the AWGS 2019 criteria, possible sarcopenia is identified by decreased muscle strength (grip strength <28 kg for males and <18 kg for females), with or without impaired physical performance (time to complete five chair stands >12 s or 6-meter walking speed <1 m/s). Because eligibility in this study was based on decreased muscle strength, participants meeting the sex-specific grip-strength thresholds were classified as having possible sarcopenia.

Cognitive function was assessed with the Chinese Changsha version of the MoCA, version 7.1 (July 2010), with a score range of 0–30 (17, 18). In accordance with the instrument instructions, one point was added for participants with no more than 6 years of formal education, up to a maximum total score of 30. The operational cutoff was normal cognition at scores ≥26 and screening-defined probable MCI at scores from 18 to <26 (equivalent to integer scores of 18–25); scores <18 were excluded from the analytic cohort because the low score could reflect more severe cognitive impairment that could not be classified as probable MCI using this screening rule. Two source records had a recorded score of 25.5; because these values were below 26, they were included in the screening-defined probable-MCI group. The outcome was therefore a MoCA-based screening classification and not a clinical diagnosis of MCI.

Inclusion criteria encompassed community-dwelling adults ≥60 years meeting the AWGS 2019 criteria for possible sarcopenia and able to complete the standardized assessments. Exclusion criteria included: (1) a MoCA score <18; (2) neurological or psychiatric disorders (e.g., myasthenia gravis, amyotrophic lateral sclerosis, or Parkinson's disease); (3) sensory impairments that prevented completion of the standardized assessments; and (4) severe systemic comorbidities, including severe liver or kidney dysfunction and malignancy. All participants provided informed consent.

The original concatenated source file contained 14,520 records. A numeric MoCA total score was available for 13,773 records (94.9%); 747 records (5.1%) lacked a usable MoCA total score. Among records with a usable MoCA score, 2,649 (19.2%) scored <18 and were excluded according to the study rule, 5,856 (42.5%) scored 18 to <26 (including the two records with a recorded score of 25.5) and were classified as screening-defined probable MCI, and 5,268 (38.2%) scored ≥26 and were classified as normal cognition. These counts sum to all 13,773 records with a usable MoCA score. The sequential screening counts were 14,520 → 14,421 → 6,823 → 6,613 → 6,574 → 6,535 → 4,523 → 3,310. The corresponding exclusions were 99, 7,598, 210, 39, 39, 2,012 (225 without a usable MoCA score and 1,787 with a MoCA score <18), and 1,213. The 225 residual records without a usable MoCA score occurred after the preceding screening steps and therefore differ from the 747 missing-MoCA records in the original source file. Variable-level missingness and the complete flow are provided in Supplementary Table S5. After these possible-sarcopenia eligibility, data-quality, outcome, and complete candidate-predictor requirements were applied, 3,310 participants remained in the analytic cohort.

A total of 3,310 participants were included in the analytic cohort: 1,013 (30.6%) in the normal-cognition group and 2,297 (69.4%) in the screening-defined probable MCI group. A standardized case-report form collected 23 variables across sociodemographic, clinical, and TCM constitutional domains. Basic indicators were sex, educational level, living alone, history of drug and food allergies, smoking history, alcohol consumption history, smartphone usage, computer usage, age, systolic and diastolic blood pressure, body mass index (BMI), Pittsburgh Sleep Quality Index (PSQI) total score, Patient Health Questionnaire-9 (PHQ-9) score, and Mini-Nutritional Assessment Short Form (MNA-SF) total score. TCM indicators were qi-deficiency constitution, qi-stagnation constitution, yang-deficiency constitution, yin-deficiency constitution, blood-stasis constitution, damp-heat constitution, phlegm-dampness constitution, and balanced constitution, based on Wang Qi's constitution framework (19).

### 2.3 TCM constitution assessment

Trained study personnel administered Wang Qi's standardized Constitution in Chinese Medicine Questionnaire (CCMQ) using standardized instructions. Each item was scored on a five-point scale. For each of the nine constitution dimensions, the transformed score was calculated as [(raw score − number of items)/(number of items × 4)] × 100. A biased constitution was considered positive at a transformed score ≥40, a score of 30–39 indicated a tendency, and a score <30 was considered negative. Balanced constitution was defined by a balanced-constitution score ≥60 and scores <30 for all eight biased constitutions. When more than one biased constitution was positive, the constitution with the highest transformed score was assigned as the principal constitution. When no biased constitution was positive but more than one showed a tendency, the highest tendency score was used. Special-diathesis constitution was treated as one of the eight biased constitutions, remained eligible as the principal category, and was used as the reference category for the eight indicator variables representing the nine-level principal-constitution variable. Thus, constitution indicators were mutually exclusive for an individual participant. The questionnaire is reproduced in Supplementary Appendix S1. Because qi-deficiency constitution and damp-heat constitution were uncommon in some data subsets, their prevalence and selection stability were examined separately and interpreted cautiously.

### 2.4 Statistical analysis

Univariate analyses were first conducted, with categorical variables presented as frequencies (percentages) and compared between groups using Pearson's chi-square test; continuity correction was applied to 2-by-2 contingency tables. Continuous variables were assessed for normality using the Shapiro–Wilk test. Variables following a normal distribution were analyzed using the independent-samples t-test, while non-normally distributed variables were evaluated using the Mann–Whitney *U* test.

The analytic dataset was first divided into a training set (70%) and a locked internal validation set (30%) using stratified random sampling by screening-defined probable MCI status (random seed 42). All data-dependent preprocessing was confined to the training data. Binary indicators were encoded as 0/1; multi-category variables were one-hot encoded with the first category omitted; and continuous variables were standardized using means and standard deviations estimated in the training data. The same fitted encoders and scaling parameters were then applied without refitting to the internal and external validation data. The modeling extract used complete candidate-predictor records after prespecified data-quality screening; no model-based imputation was applied. The complete-case approach and source-data outcome accounting are described in the Supplementary methods; model performance should therefore be interpreted for participants with complete candidate-predictor data.

LASSO regression was performed exclusively within the training data to identify a parsimonious feature set. The candidate penalty sequence comprised 200 logarithmically spaced values from 10^−4^ to 1, with a maximum of 10,000 iterations. The penalty parameter was selected by stratified 10-fold cross-validation within the training set using the one-standard-error rule; the selected value was λ = 0.026. The selected features were then supplied to six machine-learning classification models: MLP (20), GBDT (21), support vector machine (SVM) (22), K-nearest neighbors (KNN) (23), logistic regression (24), and AdaBoost (25). Ten-fold cross-validation was restricted to training-set resampling and stability assessment; the mean validation-fold ROC AUC was not treated as an independent performance validation, and an optimism-corrected AUC was not separately estimated. The available workflow did not provide a fully nested estimate in which every preprocessing and feature-selection step was re-estimated within every cross-validation fold. Each final model was fitted to the full training set using prespecified settings and evaluated once in the locked internal validation set. No oversampling, undersampling, class weighting, or other class-imbalance correction was applied; stratification was used to preserve outcome proportions. The primary MLP used one hidden layer with 100 units, rectified linear-unit activation, the Adam optimizer, an initial learning rate of 0.001, L2 penalty parameter 0.0001, automatic batch size, a maximum of 1,000 iterations, no early stopping, and random seed 42. Exact fixed settings for the five comparator algorithms are listed in Supplementary methods; no grid or randomized hyperparameter search was used for the reported six-model comparison.

Discrimination and threshold-based classification were summarized by ROC AUC, accuracy, sensitivity, specificity, and F1-score (26). Paired bootstrap resampling was used to estimate confidence intervals for differences between the MLP AUC and each comparator AUC. Calibration was summarized by the Brier score, calibration intercept, and calibration slope in both internal and external validation (27). DCA quantified potential net benefit across a range of threshold probabilities (28); no threshold was designated as a recommended clinical cutoff. SHAP values described the direction and magnitude of feature contributions to the fitted model output and were not interpreted as causal effects or independent etiological associations (29, 30).

External validation was performed in an independent sample of 314 participants (245 with screening-defined probable MCI and 69 with normal cognition) from a single institution, Henan University of Chinese Medicine, collected under the same protocol and not used in preprocessing, feature selection, training-set resampling, or model fitting. Mean grip strength in the external cohort was 15.83 ± 6.98 kg. Cohort characteristics are compared in Supplementary Tables S3–S5. Because the external data came from one institution and were relatively small, this evaluation was interpreted as limited external validation rather than proof of generalizability across regions or health-care systems. Sensitivity analyses forced age into the predictor set, excluded smartphone usage, repeated the workflow across five random seeds, and compared a conventional model without TCM constitution indicators with an extended model containing those indicators. Core machine-learning analyses were conducted using Python 3.8.10 with pandas 2.0.3, NumPy 1.24.4, scikit-learn 1.3.2, matplotlib 3.7.5, and SHAP 0.44.1; ancillary analyses used R 3.6.3. Statistical significance was set at P <0.05. Reporting was revised with reference to TRIPOD+AI (31); this reporting guideline was used to improve transparency and was not treated as evidence that the model had been prospectively or comprehensively validated.

## 3 Results

### 3.1 Group comparisons

A total of 3,310 older adults with possible sarcopenia were included, of whom 2,297 (69.38%) met the MoCA-based definition of screening-defined probable MCI and 1,013 (30.62%) were classified as having normal cognition. Among the 23 analyzed features, statistically significant between-group differences (P <0.05) were observed for sex, educational level, history of drug and food allergies, smartphone usage, computer usage, PSQI score, PHQ-9 score, balanced constitution, and damp-heat constitution (P = 0.011). The high proportion with probable MCI reflects the selected tertiary-hospital sample of older adults with possible sarcopenia and should not be interpreted as population prevalence.

Table 1 summarizes the characteristics of the study participants.

**Table 1:** Characteristics of the study participants.

Variables	Normal cognition (*N* = 1,013), *n* (%)	Probable MCI (*N* = 2,297), *n* (%)	*P*-value
Sex			<0.001
Male	690 (68.1)	1,304 (56.8)	
Female	323 (31.9)	993 (43.2)	
Educational level			<0.001
Low	482 (47.6)	1,437 (62.6)	
Middle	260 (25.7)	494 (21.5)	
High	271 (26.8)	366 (15.9)	
Living alone			0.486
Yes	80 (7.9)	164 (7.1)	
No	933 (92.1)	2,133 (92.9)	
History of drug and food allergies			0.025
Yes	57 (5.6)	181 (7.9)	
No	956 (94.4)	2,116 (92.1)	
Smoking history			0.145
Yes	199 (19.6)	401 (17.5)	
No	814 (80.4)	1,896 (82.5)	
Alcohol consumption history			0.139
Yes	185 (18.3)	370 (16.1)	
No	828 (81.7)	1927 (83.9)	
Smartphone usage			<0.001
Yes	637 (62.9)	1,217 (53.0)	
No	376 (37.1)	1,080 (47.0)	
Computer usage			<0.001
Yes	78 (7.7)	51 (2.2)	
No	935 (92.3)	2,246 (97.8)	
Age	70.23 ± 7.39	70.72 ± 7.78	0.467
Systolic blood pressure	135.03 ± 17.43	134.36 ± 19.05	0.180
Diastolic blood pressure	79.96 ± 11.16	80.48 ± 11.33	0.195
BMI	23.30 ± 2.98	23.32 ± 3.04	0.703
PSQI	6.40 ± 3.36	6.80 ± 3.65	0.002
PHQ-9	1.92 ± 3.03	2.29 ± 3.34	0.003
MNA-SF	12.18 ± 1.81	12.20 ± 1.90	0.194
Balanced constitution	268 (26.5)	689 (30.0)	0.042
Qi-deficiency constitution	44 (4.3)	128 (5.6)	0.167
Yang-deficiency constitution	102 (10.1)	275 (12.0)	0.126
Yin-deficiency constitution	93 (9.2)	236 (10.3)	0.365
Phlegm-dampness constitution	140 (13.8)	376 (16.4)	0.070
Damp-heat constitution	26 (2.6)	29 (1.3)	0.011
Blood-stasis constitution	22 (2.2)	53 (2.3)	0.909
Qi-stagnation constitution	26 (2.6)	53 (2.3)	0.744

### 3.2 Feature selection

The reported LASSO analysis retained seven features from the initial 23 variables for cross-sectional probable-MCI stratification: educational attainment, smartphone usage, sex, damp-heat constitution, qi-deficiency constitution, PSQI score, and PHQ-9 score (Figure 1). The selected penalty was the largest value within one standard error of the minimum training-fold cross-validated binomial deviance, with λ = 0.026. Age was not retained in the reported feature set; this does not imply that age is unimportant for cognitive health. In the sensitivity model that forced age into the predictor set, the internal-validation AUC increased from 0.853 to 0.877 (+0.024), while the Brier score changed from 0.168 to 0.166, the calibration intercept from −0.07 to −0.152, and the calibration slope from 0.80 to 0.808. Excluding smartphone usage produced an internal-validation AUC of 0.843, a decrease of 0.010, and did not change the remaining retained feature set. In the training set, Spearman correlations between age and educational attainment, smartphone usage, sex, PSQI, PHQ-9, qi-deficiency constitution, and damp-heat constitution were ρ = −0.005, −0.206, 0.050, 0.046, 0.086, 0.053, and 0.034, respectively. These correlations were weak, with the largest absolute value observed for smartphone usage. These data-specific changes suggest that smartphone usage contributed additional cross-sectional classification information, whereas age improved discrimination when forced into the model, but they do not establish a causal or biological hierarchy among the variables.

**Figure 1:**
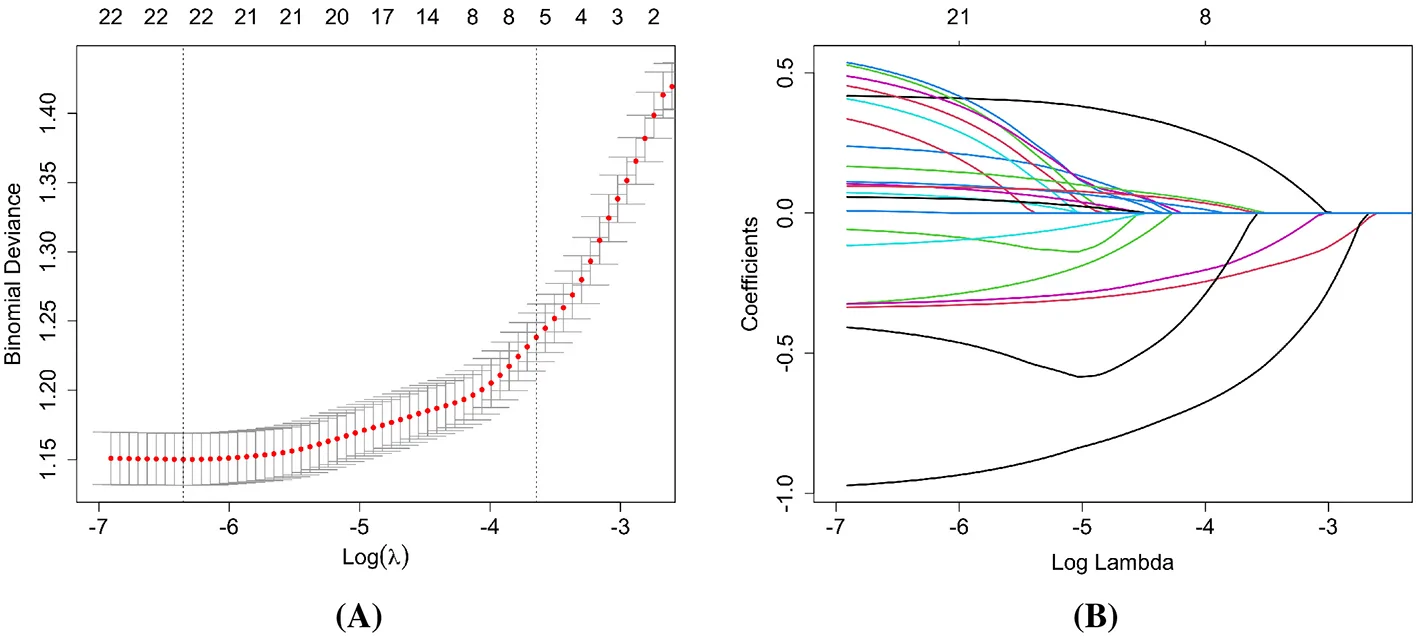
LASSO regression model selection and coefficient shrinkage. **(A)** Ten-fold cross-validation curve showing binomial deviance vs. log(λ). **(B)** LASSO coefficient profiles as a function of log(λ).

### 3.3 Construction and evaluation of the MCI risk stratification model

ROC curves were constructed to evaluate the six cross-sectional probable-MCI risk-stratification models in the internal and external validation samples (Figure 2). Each model used the seven features retained through training-set LASSO. In internal validation, the MLP showed the highest discrimination, with an AUC of 0.853 [95% confidence interval [CI]: 0.827–0.878] (Table 2). The comparator AUCs were 0.807 for GBDT, 0.807 for SVM, 0.796 for KNN, 0.740 for logistic regression, and 0.749 for AdaBoost. Paired bootstrap differences favored MLP: 0.046 (95% CI: 0.02–0.07) vs. GBDT, 0.046 (0.02–0.07) vs. SVM, 0.057 (0.03–0.08) vs. KNN, 0.113 (0.08–0.14) vs. logistic regression, and 0.104 (0.07–0.13) vs. AdaBoost. The MLP achieved an accuracy of 0.791, sensitivity of 0.819, specificity of 0.729, and F1-score of 0.845. Its mean training-set 10-fold cross-validation ROC AUC was 0.874, with a standard deviation of 0.024 and a fold-level range of 0.839–0.908; this value is reported as a training-set resampling/stability estimate and not as a separate validation result or an optimism-corrected estimate. Across five random seeds, the internal-validation AUC ranged from 0.847 to 0.861, with a mean of 0.856 and standard deviation of 0.006, indicating limited variation in discrimination across seeds.

**Figure 2:**
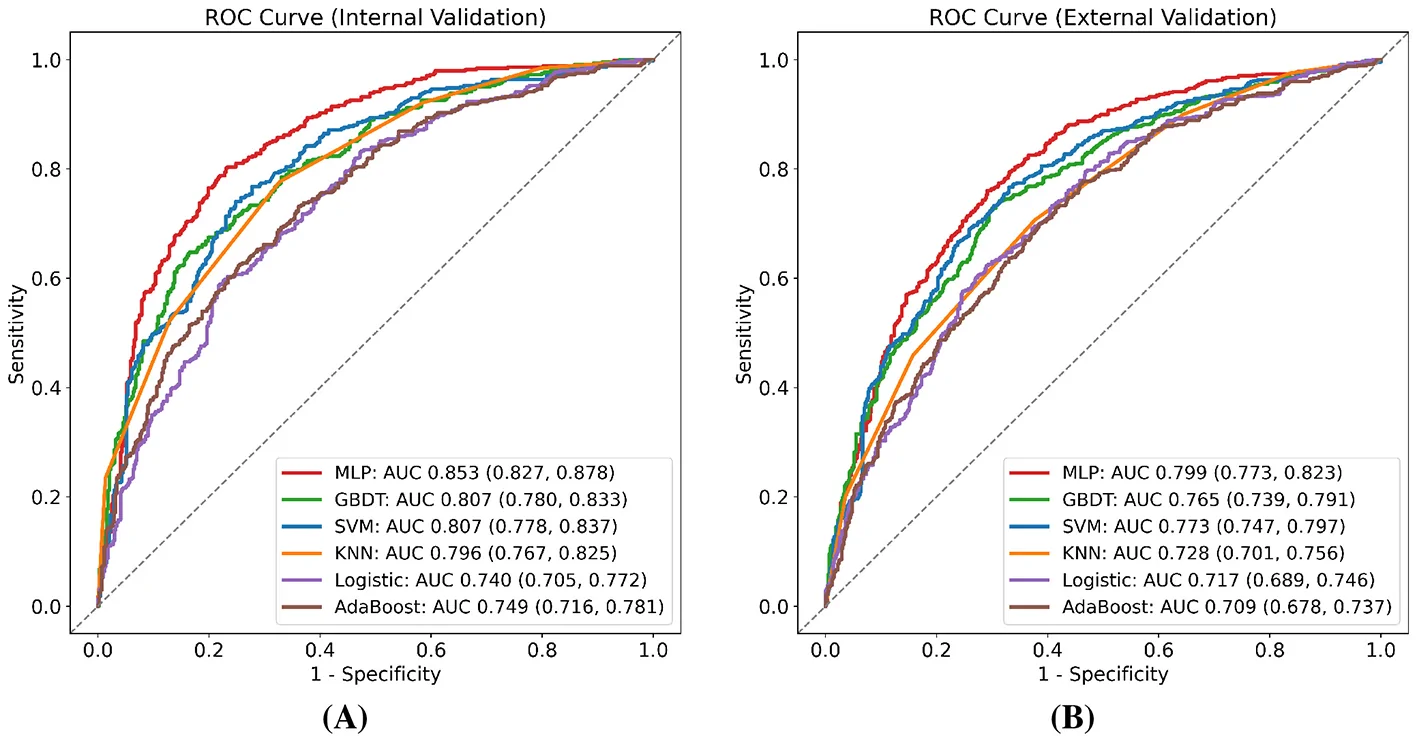
ROC curves of six machine-learning risk stratification models. **(A)** Internal validation. **(B)** External validation.

**Table 2:** Performance of the machine-learning models in internal validation.

Model	AUC	Accuracy	Sensitivity	Specificity	F1-Score	10-Fold CV
MLP	0.853	0.791	0.819	0.729	0.845	0.874
GBDT	0.807	0.739	0.767	0.677	0.803	0.861
SVM	0.807	0.751	0.777	0.691	0.812	0.856
KNN	0.796	0.745	0.777	0.672	0.809	0.858
Logistic regression	0.740	0.706	0.754	0.598	0.781	0.813
AdaBoost	0.749	0.703	0.731	0.638	0.773	0.815

In a sensitivity analysis using an alternative screening definition, participants with MoCA scores of 18–21 were classified as screening-defined probable MCI and those with scores ≥22 as cognitively normal, while the prespecified exclusion of scores <18 was retained. This definition classified 1,364 participants as probable MCI. Using the same stratified 70:30 split, the MLP achieved an internal-validation AUC of 0.804 (95% CI: 0.772–0.834), accuracy of 0.758, sensitivity of 0.780, specificity of 0.692, and F1-score of 0.719. The Brier score was 0.222, with a calibration intercept of −0.277 and a calibration slope of 0.552. Discrimination remained acceptable, although calibration was less favorable than under the primary outcome definition.

In the pre-specified incremental-value analysis, the conventional MLP model without TCM constitution indicators had an AUC of 0.685, accuracy of 0.640, sensitivity of 0.748, specificity of 0.522, and F1-score of 0.686. The extended MLP model had an AUC of 0.853, accuracy of 0.791, sensitivity of 0.819, specificity of 0.729, and F1-score of 0.845. Both models used the same locked internal split, outcome observations, preprocessing framework, and MLP architecture. The paired difference in AUC was 0.166 (95% CI: 0.135–0.196), calculated from paired unrounded predictions and therefore not necessarily equal to subtraction of the displayed AUCs rounded to three decimals. The Brier scores were approximately 0.176 for the conventional model and 0.168 for the extended model; the paired difference was −0.008 (95% CI: −0.013 to −0.003), favoring the extended model. The conventional model had an internal calibration intercept of approximately 0.00 and slope of 1.17, compared with −0.07 and 0.80 for the extended model. DCA was evaluated over prespecified threshold probabilities from 0.30 to 0.70. The net benefits for the conventional and extended models were 0.56 and 0.58 at 0.30 (difference 0.02), 0.48 and 0.53 at 0.40 (difference 0.05), 0.37 and 0.48 at 0.50 (difference 0.11), 0.27 and 0.39 at 0.60 (difference 0.12), and 0.14 and 0.25 at 0.70 (difference 0.11). The corresponding treat-all net benefits were 0.563, 0.490, 0.388, 0.235, and −0.020, respectively; treat-none net benefit was 0 at every threshold. Model net-benefit values are reported to two decimal places and treat-all values to three decimal places. These DCA results are interpreted as potential net benefit, not as recommended clinical cutoffs. Because this comparison used the same internal sample and the external incremental-value analysis was not performed, it supports a cohort-specific incremental contribution rather than established clinical utility.

In the independent single-institution external validation sample (n = 314; 245 with screening-defined probable MCI and 69 with normal cognition), the MLP AUC was 0.799 (95% CI: 0.773–0.823), lower than the internal validation AUC of 0.853. The internal Brier score, calibration intercept, and calibration slope were 0.168, −0.07, and 0.80, respectively; the corresponding external values were 0.151, 0.18, and 1.07. The external intercept indicates modest overall underestimation of the observed outcome probability, while the slope suggests comparatively mild underfitting. Differences in sample size, probable-MCI proportion, single-institution recruitment, education, and predictor/constitution distributions may have contributed to the lower external discrimination. Additional performance measures and cohort characteristics are presented in Supplementary Tables S2–S5.

Figure 3 presents calibration and clinical-utility analyses. Calibration curves for five models (GBDT, SVM, KNN, logistic regression, and MLP) are shown in Figure 3A; AdaBoost is not included in this panel. In DCA (Figure 3C), both the conventional and extended models showed potential net benefit relative to treat-all and treat-none strategies over parts of the prespecified threshold range. The extended model had higher net benefit than the conventional model at each reported threshold: 0.58 vs. 0.56 at 0.30, 0.53 vs. 0.48 at 0.40, 0.48 vs. 0.37 at 0.50, 0.39 vs. 0.27 at 0.60, and 0.25 vs. 0.14 at 0.70. The corresponding differences were 0.02, 0.05, 0.11, 0.12, and 0.11. These results describe potential net benefit across threshold probabilities and do not define a recommended clinical cutoff. Model selection therefore considered DCA together with calibration, discrimination, and model complexity rather than relying on AUC alone.

**Figure 3:**
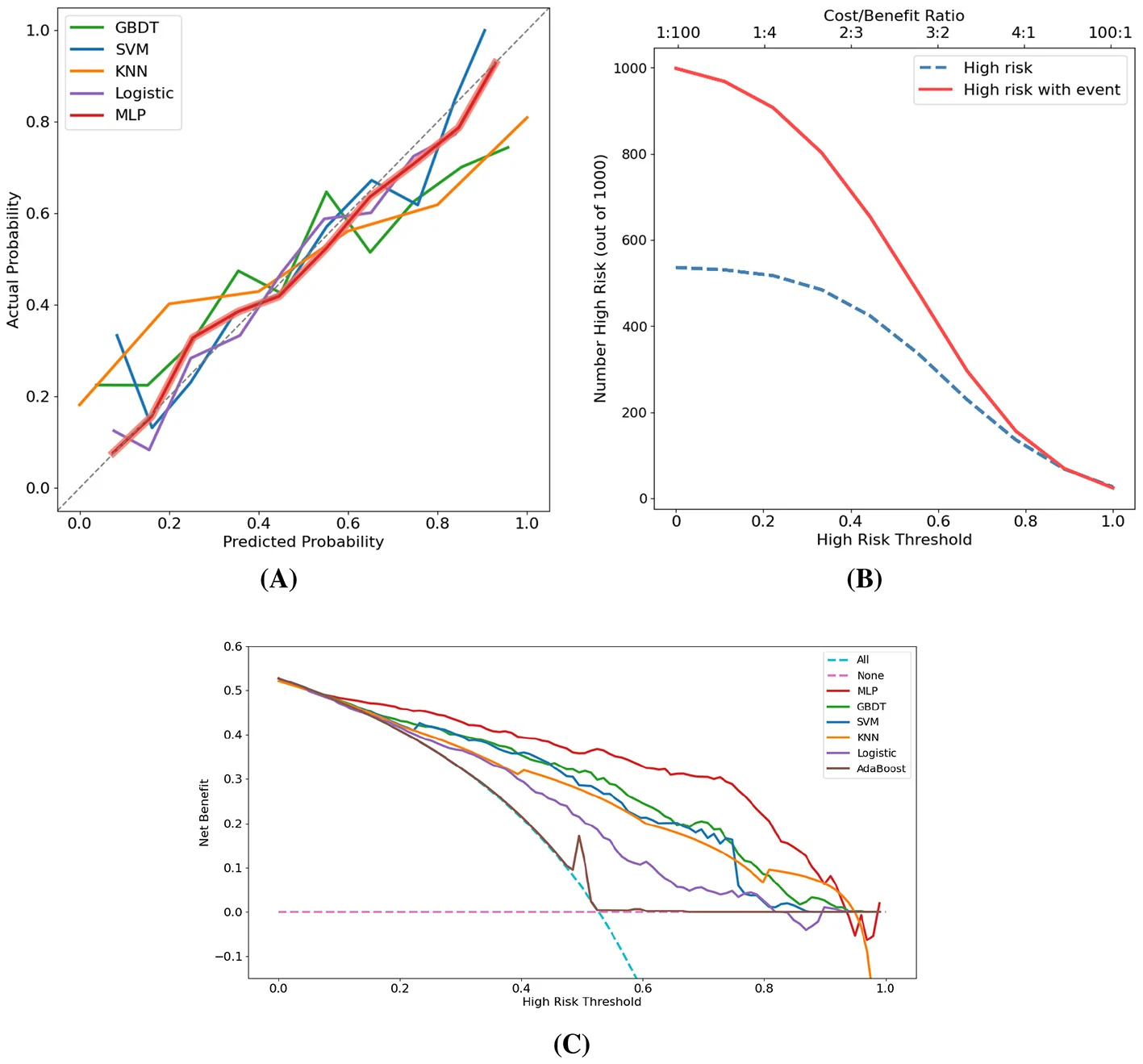
Calibration and clinical-utility analyses of the risk-stratification models. **(A)** Calibration curves showing agreement between model-estimated probabilities and observed screening-defined probable-MCI status. **(B)** Clinical impact curve (CIC); convergence near 0.7 is descriptive and does not define a recommended cutoff. **(C)** Decision curve analysis (DCA) showing model-specific potential net benefit across the evaluated probability thresholds.

Clinical impact curves (CIC; Figure 3B) were generated for a hypothetical sample of 1,000 older adults. The x-axis represents probability thresholds, and the left and right y-axes show the numbers classified as high probability and the observed probable-MCI cases among those classified as high probability. The convergence seen around 0.7 is descriptive only. It does not establish 0.7 as a clinical cutoff; any operational threshold would require a prespecified referral action, explicit weighting of false positives and false negatives, and prospective validation in the intended screening setting.

### 3.4 SHAP analysis

SHAP analysis ranked contributions to the fitted MLP output as follows: educational attainment > sex > PHQ-9 score > PSQI score > smartphone usage > qi-deficiency constitution > damp-heat constitution (Figure 4A). Figure 4B shows how feature values shifted the model-assigned probability of screening-defined probable MCI in this dataset. These SHAP values explain the fitted model's output; they do not establish causal effects, independent risk factors, or effects of intervention.

**Figure 4:**
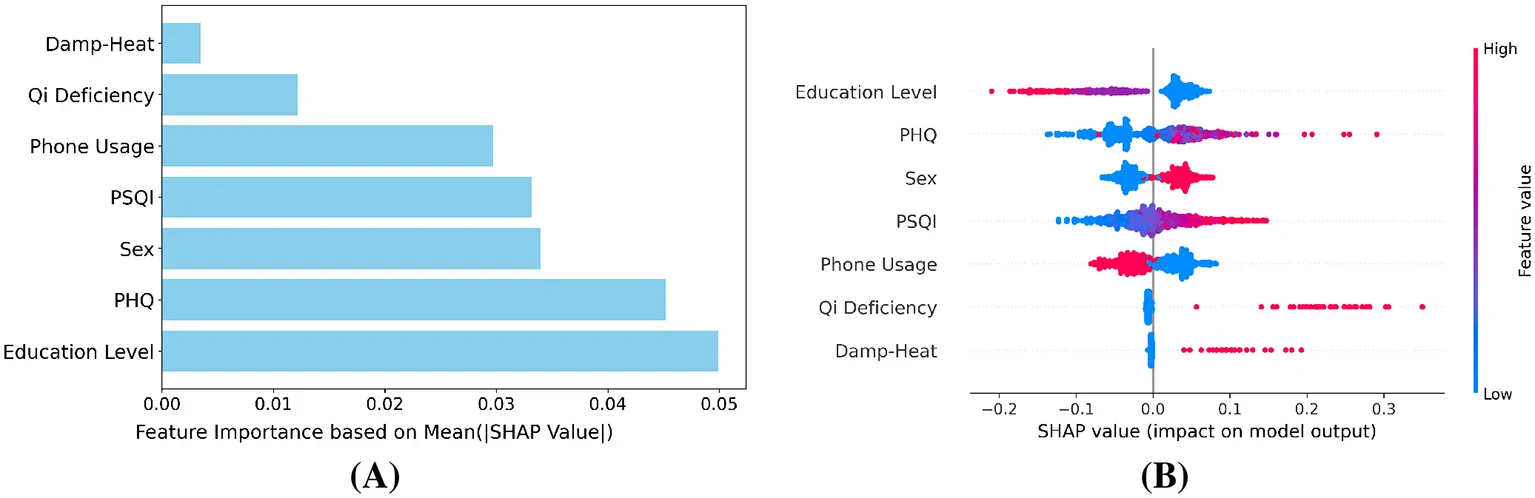
Feature importance and SHAP values for model interpretation. **(A)** Relative feature importance based on mean absolute SHAP values. **(B)** Associations between feature values and model output.

## 4 Discussion

This cross-sectional study identified sex, educational attainment, smartphone usage, sleep quality, depressive symptoms, and TCM constitution categories as features contributing to stratification of current screening-defined probable MCI among older adults with possible sarcopenia. The findings describe concurrent associations and model-output contributions, not predictors of future incident MCI. The proportion classified as having probable MCI was higher among women than among men [75.5% [993/1,316] vs. 65.4% [1,304/1,994]; *P* <0.001], consistent with population-based evidence from older Chinese adults showing a higher age-standardized prevalence of MCI among women (32). Hormonal and reproductive mechanisms have been proposed in the broader literature (33, 34), but they were not measured here and cannot explain the present cross-sectional association.

The overall probable-MCI proportion of 69.4% is higher than population-based estimates and should not be generalized to community prevalence. Recruitment from tertiary hospitals and restriction to older adults with possible sarcopenia enriched the sample for physical vulnerability and may also have increased the proportion with low MoCA scores. In addition, the outcome was defined by a screening rule rather than adjudicated clinical diagnosis. These design features likely contributed to the high observed proportion.

Low educational attainment was retained as an important feature in the risk-stratification model. Prior evidence supports an association between education and cognitive function, often interpreted through the concept of cognitive reserve. Within the present cross-sectional sample, lower educational attainment was associated with concurrent screening-defined probable-MCI status, but the study design does not establish that education or muscle–brain axis dysfunction caused the observed difference.

Participants who used smartphones or computers were less likely to be classified as having probable MCI in the univariate comparisons, and smartphone usage was retained by LASSO regression. Excluding smartphone usage reduced internal AUC from 0.853 to 0.843 (0.010, or 1.0 percentage point), while the remaining retained feature set did not change. This indicates that smartphone usage carried additional cross-sectional classification information in this dataset. It does not demonstrate that digital device use prevents cognitive impairment: better current cognitive or functional status may enable device use, and access and socioeconomic factors may confound the association. A nationwide cross-sectional study likewise reported associations between multidimensional internet use and cognitive performance (35). Smartphone usage should therefore be viewed as a concurrent screening feature rather than a modifiable causal factor.

Higher PSQI and PHQ-9 scores contributed to a higher model-assigned probability of concurrent screening-defined probable MCI. Previous cross-sectional research has reported associations of sleep disorders and fatigue with poorer cognitive function among older adults (36). Longitudinal research has also linked depression with subsequent dementia risk (37), while a meta-analysis identified overlapping structural brain alterations in cognitive impairment and depression (38). These studies provide context but do not establish the direction or mechanisms of the relationships observed in the present cross-sectional sample.

Qi-deficiency constitution and damp-heat constitution were retained in the MLP and contributed to its model-assigned probability according to SHAP. Within the TCM constitution framework, these categories are intended to capture individualized functional and physiological patterns that may co-occur with differences in sleep, mood, nutrition, or physical functioning relevant to cognitive screening. This provides a conceptual rationale for evaluating them as accessible screening features, not evidence of a biological mechanism or causal effect. Previous cross-sectional studies have reported associations involving other TCM constitution categories and cognitive scores (39, 40). In the 500-resample training-set stability analysis, damp-heat constitution was selected in 493 resamples (98.6%), whereas qi-deficiency constitution was selected in 169 (33.8%). Thus, damp-heat constitution showed high selection stability, while the qi-deficiency result was less stable and requires confirmation in larger independent cohorts. The conventional-vs.-extended comparison suggested improved internal discrimination and Brier score after adding TCM constitution, but incremental value was not tested in the external sample. The findings therefore support a cohort-specific contribution that requires independent replication; they do not establish causal effects or intervention targets.

From a public-health perspective, TCM constitution may be evaluated as an accessible feature in individualized screening pathways and may inform the design of future preventive studies. Prior research has explored exercise, herbal, and acupuncture interventions in cognitive frailty, experimental Alzheimer disease, or MCI (41–43). These studies represent future intervention opportunities only; the present study neither tested an intervention nor established a TCM intervention strategy.

The MLP was retained as the primary research model because it showed the highest internal AUC and statistically higher paired AUCs than the five comparators. Nevertheless, selection of a model for implementation cannot rely on AUC alone. Its internal calibration slope of 0.80, threshold-dependent DCA findings, computational complexity, and the feasibility of collecting its inputs must be weighed against simpler alternatives. If future prospective evaluation shows comparable calibration and net benefit for a simpler model, that model may be preferable for routine use.

The decrease in MLP AUC from 0.853 internally to 0.799 externally is compatible with transportability loss. Potential contributors include the smaller external sample, a different probable-MCI proportion, recruitment from one institution, and differences in education, clinical characteristics, smartphone use, and constitution distributions. The external calibration intercept of 0.18 also suggests modest underestimation of outcome probability. Recalibration may therefore be needed before application in another setting, but no recalibration was performed or claimed in this study. With only 314 participants from one institution, the external analysis supports limited validation in a related setting and does not demonstrate generalizability across regions or health-care systems.

Recent longitudinal studies illustrate the temporal distinction that the present design cannot address. Long et al. used repeated CHARLS waves to predict a subsequent depression outcome and evaluated the model in CLHLS (44); Chalmers et al. used baseline data to model 10-year incident dementia in the Sydney Memory and Ageing Study (45). In contrast, the present variables and MoCA status were assessed concurrently. Its contribution is therefore an integrative cross-sectional screening framework, not a model of incident MCI or future cognitive decline.

## 5 Limitations

This study has several limitations. First, its cross-sectional design precludes causal inference and cannot estimate incident MCI or future cognitive decline. Second, the outcome was screening-defined probable MCI based on the operational MoCA rule rather than a clinical diagnosis; participants scoring below 18 were excluded, and outcome misclassification remains possible. No clinician-adjudicated reference standard was available, so sensitivity and specificity of the study cutoff against a clinical diagnosis could not be estimated. Although the Changsha MoCA version has been validated as a cognitive-screening instrument in another Chinese clinical population (18), the present 18-to-below-26 grouping, including the two source records with a score of 25.5, and exclusion of scores below 18 were operational study rules and were not separately validated in older adults with possible sarcopenia. An alternative screening definition using 18–21 vs. ≥22 was examined and yielded an AUC of 0.804, but its calibration was less favorable than that of the primary definition; education-stratified thresholds were not evaluated. Third, tertiary-hospital recruitment and restriction to possible sarcopenia likely enriched the sample and contributed to the high probable-MCI proportion, limiting applicability to community populations. Fourth, the external cohort contained only 314 participants from one institution. It therefore provides limited external validation and cannot establish transportability across regions, cultures, or health-care systems. Fifth, the external calibration intercept indicates that local recalibration may be required, but recalibration was not performed. Sixth, self-reported sleep, depressive symptoms, and digital-device use may be affected by reporting bias and reverse causation. Seventh, variable-level missingness is summarized in Supplementary Table S5, but the complete-case analysis may still introduce selection bias if missingness was related to outcome or predictors. Eighth, the 500-resample selection frequencies for qi-deficiency constitution and damp-heat constitution, the age correlations with retained variables, and the fold-level dispersion of the training-set 10-fold resampling AUC are reported; however, the entire preprocessing and feature-selection pipeline was not evaluated in a fully nested cross-validation design, and an optimism-corrected AUC was not separately estimated. Finally, APOE genotype, laboratory biomarkers, and other potentially informative variables were unavailable. The framework should be evaluated as one component of a multi-step screening pathway followed by standardized cognitive assessment, not as a standalone diagnostic tool.

## 6 Conclusions

This study developed an interpretable cross-sectional machine-learning framework for stratifying the probability of current screening-defined probable MCI among older adults with possible sarcopenia. In the prespecified internal conventional-vs.-extended comparison, including TCM constitution indicators was associated with higher discrimination and lower Brier error; its incremental value requires external confirmation. The single-institution external evaluation supports only limited transportability. Prospective multicenter studies should evaluate calibration, operational thresholds, and clinical consequences before the framework is considered for use in a multi-step screening pathway.

## Data Availability

The datasets are not publicly available because they contain potentially identifiable participant information and are subject to institutional ethical restrictions. De-identified data may be made available by the corresponding author upon reasonable request and with approval from the relevant ethics committee.
