# Supplementary material for: Development and validation of a risk stratification model for mild cognitive impairment among older adults with possible sarcopenia integrating traditional Chinese medicine constitution: a multicenter cross-sectional study
Source: Front Public Health. 2026 Oct 30;14:1980675. doi: 10.3389/fpubh.2026.1980675 (PMC13759270; doi:10.3389/fpubh.2026.1980675)
Supplement: Supplementary file 1 [file Data_Sheet_1.pdf]

Supplementary Material

SUPPLEMENTARY METHODS

Outcome definition. Cognitive status was assessed with the Chinese Changsha version of the Montreal Cognitive Assessment (MoCA), version 7.1 (July 2010; score range 0–30). One point was added for participants with no more than six years of formal education, up to a maximum score of 30. Scores $\geq$26 defined normal cognition, scores from 18 to $<$26 (equivalent to integer scores of 18–25) defined screening-defined probable MCI, and scores $<$18 were excluded. Two source records had a recorded score of 25.5; because these values were below 26, they were included in the screening-defined probable-MCI group. This operational rule preserves the original integer cutoffs and was not intended to constitute a clinical diagnosis.

Source-data outcome flow. The original concatenated source file contained 14,520 records. A numeric MoCA total score was available for 13,773 records (94.9%); 747 records (5.1%) lacked a usable MoCA total score. Among records with a usable MoCA score, 2,649 (19.2%) scored $<$18 and were excluded according to the study rule, 5,856 (42.5%) scored 18 to $<$26 (including the two records with a recorded score of 25.5) and were classified as screening-defined probable MCI, and 5,268 (38.2%) scored $\geq$26 and were classified as normal cognition. These counts sum to all 13,773 records with a usable MoCA score. The sequential screening flow and the denominator used for variable-level missingness are provided in Supplementary Table S5. An alternative screening definition was also examined: scores of 18–21 were classified as screening-defined probable MCI and scores $\geq$22 as normal cognition, while scores $<$18 remained excluded. Education-stratified alternative thresholds were not evaluated.

Analysis sequence and leakage control. The multicenter analytic cohort was divided by stratified random sampling into a 70% training set and a 30% locked internal validation set using random seed 42. Missing-data screening, category mapping, one-hot encoding, standardization, and LASSO feature selection were performed using the training data only. Continuous-variable means and standard deviations and all category maps were stored and applied unchanged to internal and external validation. The external cohort (n=314) was not used in any model-development decision. Ten-fold stratified cross-validation was confined to the training set for penalty selection and resampling-based stability assessment. The cross-validation mean AUC was not treated as an independent validation estimate, and the available analysis did not provide an optimism-corrected AUC or a fully nested estimate of the complete preprocessing and feature-selection pipeline. No oversampling, undersampling, class weighting, or other class-imbalance correction was applied; stratification was used to preserve outcome proportions. The final models were refitted using the full training set with prespecified fixed settings and then evaluated once in the locked internal validation set and once in the external dataset.

Missing data and preprocessing. Candidate records underwent prespecified range and completeness checks before modeling. Variable-level missingness and the sequential exclusion flow are reported in Supplementary Table S5. No model-based imputation was performed; the final modeling matrix used complete records for the included candidate variables. The source-level MoCA accounting (747 records without a usable MoCA score) and the residual 225 records without a usable MoCA score at the outcome-screening stage use different denominators and are both shown in Supplementary Table S5. Binary variables were encoded as 0/1. Educational level and other multi-category variables were one-hot encoded with the first category omitted. Continuous variables were standardized using the training-set mean and standard deviation. No encoder, scaler, imputation rule, or feature-selection parameter was estimated from the internal or external validation data.

TCM constitution variables. Trained personnel administered Wang Qi's standardized Constitution in Chinese Medicine Questionnaire (CCMQ) using standardized instructions. Each item was scored on a five-point scale. For each of the nine constitution dimensions, the transformed score was calculated as $[(\text{raw score} - \text{number of items})/(\text{number of items} \times 4)] \times 100$. A biased constitution was considered positive at a transformed score $\geq$40, a score of 30–39 indicated a tendency, and a score $<$30 was considered negative. Balanced constitution was defined by a balanced-constitution score $\geq$60 and scores $<$30 for all eight biased constitutions. When more than one biased constitution was positive, the constitution with the highest transformed score was assigned as the principal constitution. When no biased constitution was

positive but more than one showed a tendency, the highest tendency score was used. Special-diathesis constitution was treated as one of the eight biased constitutions, remained eligible as the principal category, and served as the reference category for the eight indicator variables representing the nine-level principal-constitution variable. Thus, constitution indicators were mutually exclusive for each participant. The prevalence of qi-deficiency constitution and damp-heat constitution in the training, internal-validation, and external-validation sets is shown in Supplementary Table S4.

Feature selection and model specification. LASSO was performed within the training set using 200 logarithmically spaced penalty values from 10^{-4} to 1 and a maximum of 10,000 iterations. The penalty parameter was selected by stratified 10-fold cross-validation within the training set using the one-standard-error rule; the selected value was $\lambda = 0.026$. The reported classifiers used fixed settings rather than a grid or randomized hyperparameter search. The MLP used one hidden layer with 100 units, rectified linear-unit activation, Adam optimization, an initial learning rate of 0.001, L2 penalty parameter 0.0001, automatic batch size, a maximum of 1,000 iterations, no early stopping, and random seed 42. GBDT used 100 estimators, learning rate 0.1, maximum tree depth 3, and random seed 42. SVM used a linear kernel, $C = 1.0$, probability estimation enabled, and random seed 42. KNN used five neighbors, uniform weighting, and Minkowski distance with $p = 2$. Logistic regression used L2 regularization, $C = 1.0$, the lbfgs solver, and a maximum of 10,000 iterations. AdaBoost used a decision stump (maximum depth 1) as the base estimator, two estimators, learning rate 1.0, the SAMME.R algorithm, and random seed 42. XGBoost, Gaussian naive Bayes, random forest, and LightGBM scripts were exploratory and were not included in the reported six-model comparison. **Performance and sensitivity analyses.** ROC AUC, accuracy, sensitivity, specificity, and F1-score were calculated in the locked internal validation set. Brier score, calibration intercept, and calibration slope were reported for the MLP in internal and external validation. DCA was evaluated over prespecified threshold probabilities and interpreted as potential net benefit; the clinical impact curve was descriptive and did not establish a recommended cutoff. Across thresholds of 0.30, 0.40, 0.50, 0.60, and 0.70, the net benefits for the conventional and extended MLPs were 0.56 versus 0.58, 0.48 versus 0.53, 0.37 versus 0.48, 0.27 versus 0.39, and 0.14 versus 0.25, respectively. The corresponding model differences were 0.02, 0.05, 0.11, 0.12, and 0.11; treat-all net benefits were 0.563, 0.490, 0.388, 0.235, and -0.020, respectively, and treat-none net benefit was 0 at every threshold. Model net-benefit values are reported to two decimal places and treat-all values to three decimal places. Paired bootstrap resampling generated 95% confidence intervals for AUC differences. The 10-fold cross-validation AUC was treated as a training-set resampling estimate; its mean was 0.874, with a standard deviation of 0.024 and a fold-level range of 0.839–0.908. Sensitivity analyses forced age into the predictor set, excluded smartphone usage, repeated the workflow with five random seeds, and compared a conventional model without TCM constitution indicators against an extended model including TCM indicators. The age-forced model had an internal-validation AUC of 0.877, Brier score of 0.166, calibration intercept of -0.152, and calibration slope of 0.808, compared with 0.853, 0.168, -0.07, and 0.80, respectively, for the seven-variable primary model. Excluding smartphone usage yielded an AUC of 0.843 and did not change the remaining retained feature set. Across five random seeds, the internal-validation AUC ranged from 0.847 to 0.861 (mean 0.856, SD 0.006). The five-seed analysis assessed discrimination stability, whereas a separate 500-resample training-set bootstrap assessed feature-selection stability: damp-heat constitution was selected in 493/500 resamples (98.6%) and qi-deficiency constitution in 169/500 (33.8%). Spearman correlations between age and the seven retained predictors are reported in the main text. SHAP values were used to interpret fitted-model output and not causal effects.

Software and reporting. Core machine-learning scripts were run under Python 3.8.10 with pandas 2.0.3, NumPy 1.24.4, scikit-learn 1.3.2, matplotlib 3.7.5, and SHAP 0.44.1. Ancillary analyses used R 3.6.3. Reporting was revised with reference to TRIPOD+AI; this guideline was used to improve transparency and does not itself establish methodological quality or validation.

1 SUPPLEMENTARY TABLE S1

Table S1. Participating institutions and their geographical regions.

No.	Institution	Region
1	Affiliated Hospital of Chengdu University of Traditional Chinese Medicine	Sichuan, China
2	Shuguang Hospital Affiliated to Shanghai University of Traditional Chinese Medicine	Shanghai, China
3	Guangdong Provincial Hospital of Integrated Traditional Chinese and Western Medicine	Guangdong, China
4	Longgang Hospital of Shenzhen	Guangdong, China
5	Integrated Hospital of Traditional Chinese and Western Medicine, Southern Medical University	Guangdong, China
6	Hainan Provincial Hospital of Traditional Chinese Medicine	Hainan, China
7	The Second Affiliated Hospital of Liaoning University of Traditional Chinese Medicine	Liaoning, China
8	Yunnan Provincial Hospital of Traditional Chinese Medicine	Yunnan, China
9	Affiliated Hospital of Ningxia Medical University	Ningxia, China
10	Zhejiang Provincial Hospital of Traditional Chinese Medicine	Zhejiang, China

2 SUPPLEMENTARY APPENDIX S1: TCM CONSTITUTION ASSESSMENT SCALE

The English-language version of Wang Qi's Body Constitution Classification Questionnaire used for TCM constitution assessment is reproduced below.

Wang Qi's Body Constitution Classification Questionnaire

This questionnaire aims to survey your constitution and to subsequently provide a reference for future health management and clinical diagnosis. Please read every question carefully and choose the most suitable response based on your actual situation or experience in the past year. If you are unsure of the answer to a specific question, choose the answer that is most similar to your actual situation. Make sure that you answer all the questions based on your situation **in the past year**, (excluding the effect of drugs) and give only one answer to each question.

Explain: 【No】Never happened in the past year. 【Slightly】Occasionally happened in the past year.
【Sometimes】Sometimes it happened, but no regular pattern. 【Often】It happened most of the time in the past year.
【All the time】It happened all the time in the past year.

Experience/condition in the past year	No	Slightly	Sometimes	Often	All the time
(1) Were you energetic?	1	2	3	4	5
(2) Did you get tired easily?	1	2	3	4	5
(3) Did you experience shortness of breath?	1	2	3	4	5
(4) Did you get palpitations?	1	2	3	4	5
(5) Did you get dizzy easily or become dizzy when standing up?	1	2	3	4	5
(6) Did you prefer quietness and not like to talk?	1	2	3	4	5
(7) Was your voice weak when talking?	1	2	3	4	5
(8) Did you feel in low spirits and depressed?	1	2	3	4	5
(9) Did you easily feel anxious and worried?	1	2	3	4	5
(10) Did you feel overly sensitive, vulnerable or emotionally upset?	1	2	3	4	5
(11) Were you easily scared or frightened?	1	2	3	4	5
(12) Did you experience distention in the underarm or breast?	1	2	3	4	5
(13) Did you feel chest or abdominal stuffiness?	1	2	3	4	5
(14) Did you sigh without reason?	1	2	3	4	5
(15) Did your body feel heavy or lethargic?	1	2	3	4	5
(16) Did the palms of your hands or soles of your feet feel hot ?	1	2	3	4	5
(17) Did your hands or feet feel cold or clammy?	1	2	3	4	5
(18) Did you feel cold easily in your abdomen, back, lower back or knees?	1	2	3	4	5
(19) Were you sensitive to cold and tended to wear more clothes than others?	1	2	3	4	5
(20) Did your body and face feel hot?	1	2	3	4	5
(21) Did you feel more vulnerable to the cold than others (winter coldness, air conditioners, fans, etc.)?	1	2	3	4	5
(22) Did you catch colds more easily than others?	1	2	3	4	5
(23) Did you sneeze even when you *did not* have a cold?	1	2	3	4	5
(24) Did you have a runny or stuffy nose even when you *did not* have a cold?	1	2	3	4	5
(25) Did you cough due to seasonal changes, temperature changes, or unpleasant odors?	1	2	3	4	5
(26) Did you sweat easily when your physical activity increased slightly?	1	2	3	4	5
(27) Did you forget things easily?	1	2	3	4	5
(28) Did you have an excessively oily forehead and/or T-zone?	1	2	3	4	5
(29) Were your lips redder than in the past?	1	2	3	4	5
(30) Did you have allergies? (e.g. medicine, food, odors, pollen, pet dander, or during seasonal or weather change etc.)	1	2	3	4	5

2

Figure S1: Wang Qi's Body Constitution Classification Questionnaire, page 1 of 2.

Experience/condition in the past year	No	Slightly	Sometimes	Often	All the time
(31) Did you get hives/urticaria easily?	1	2	3	4	5
(32) Did your skin have purpura (purple spots, ecchymosis) due to allergies?	1	2	3	4	5
(33) Did black or purple bruises appear on your skin for no reason?	1	2	3	4	5
(34) Did your skin turn red and show traces when you scratched it?	1	2	3	4	5
(35) Did your skin or lips feel dry?	1	2	3	4	5
(36) Did you have visible capillary (thread) veins on your cheeks?	1	2	3	4	5
(37) Did you feel pain somewhere in your body?	1	2	3	4	5
(38) Did you experience hot flashes?	1	2	3	4	5
(39) Did your nose or your face feel greasy, oily, or shiny?	1	2	3	4	5
(40) Did you have a dark face or get brown spots easily?	1	2	3	4	5
(41) Did you get acne or sores easily?	1	2	3	4	5
(42) Did you have upper eyelid swelling?	1	2	3	4	5
(43) Did you get dark circles under the eyes easily?	1	2	3	4	5
(44) Did your eyes feel dry and you used eye drops?	1	2	3	4	5
(45) Were your lips darker, more blue or purple than usual?	1	2	3	4	5
(46) Did you often feel parched and need to drink water?	1	2	3	4	5
(47) Did your throat feel strange (i.e., as if something was stuck or there was a lump in your throat)?	1	2	3	4	5
(48) Did you have a bitter or strange taste in your mouth?	1	2	3	4	5
(49) Did your mouth feel sticky?	1	2	3	4	5
(50) Was your abdomen flabby?	1	2	3	4	5
(51) Did you have an abundance of phlegm, especially in your throat?	1	2	3	4	5
(52) Did you feel uncomfortable when you drank or ate something cold, or did you avoid to drinking or eating cold items?	1	2	3	4	5
(53) Could you adapt yourself to external natural or social environment changes?	1	2	3	4	5
(54) Did you easily experience insomnia?	1	2	3	4	5
(55) Did you easily contract diarrhea when you were exposed to cold or ate (or drank) something cold?	1	2	3	4	5
(56) Did you pass sticky stools and/or feel that your bowel movement was incomplete?	1	2	3	4	5
(57) Did you get constipated easily or have dry stools?	1	2	3	4	5
(58) Did your tongue have a thick coating?	1	2	3	4	5
(59) Did your urethral canal feel hot when you urinated, or did your urine have a dark color?	1	2	3	4	5
(60) Was your vaginal discharge yellowish (**only for female interviewees**)?	1	2	3	4	5
(60) Was your scrotum always wet (**only for male interviewees**)?	1	2	3	4	5

3

Figure S2: Wang Qi's Body Constitution Classification Questionnaire, page 2 of 2.

3 SUPPLEMENTARY TABLE S2

Table S2. Performance of the machine-learning models in the external validation sample (n=314).

Model	AUC	Accuracy	Sensitivity	Specificity	F1-Score
MLP	0.799	0.755	0.774	0.688	0.831
GBDT	0.765	0.739	0.767	0.639	0.821
SVM	0.773	0.750	0.781	0.640	0.830
KNN	0.728	0.688	0.706	0.623	0.779
Logistic Regression	0.717	0.706	0.739	0.590	0.797
AdaBoost	0.709	0.686	0.711	0.596	0.779

MLP calibration summary. In internal validation, the Brier score was 0.168, the calibration intercept was -0.07, and the calibration slope was 0.80. In external validation, the corresponding values were 0.151, 0.18, and 1.07. These values were reported without recalibrating the model. The positive external intercept indicates modest overall underestimation of the observed probable-MCI probability, and the external slope suggests comparatively mild underfitting.

Paired model comparisons. Internal paired bootstrap differences in AUC for MLP versus GBDT, SVM, KNN, logistic regression, and AdaBoost were 0.046 (95% CI: 0.02–0.07), 0.046 (0.02–0.07), 0.057 (0.03–0.08), 0.113 (0.08–0.14), and 0.104 (0.07–0.13), respectively. Across five random seeds, the internal-validation AUC ranged from 0.847 to 0.861, with a mean of 0.856 and standard deviation of 0.006. This indicates limited variation in discrimination across seeds.

Incremental-value analysis. The conventional MLP model without TCM constitution indicators had AUC 0.685, accuracy 0.640, sensitivity 0.748, specificity 0.522, and F1-score 0.686. The extended MLP model had AUC 0.853, accuracy 0.791, sensitivity 0.819, specificity 0.729, and F1-score 0.845. Both models used the same locked internal split, outcome observations, preprocessing framework, and MLP architecture. The Brier scores were approximately 0.176 for the conventional model and 0.168 for the extended model. The paired AUC difference was 0.166 (95% CI: 0.135–0.196), calculated from paired unrounded predictions and therefore not necessarily equal to subtraction of the displayed AUCs rounded to three decimals. The paired Brier-score difference was -0.008 (95% CI: -0.013 to -0.003), favoring the extended model. The conventional Brier score is approximate because it was derived from the rounded extended-model Brier score and the paired difference. This comparison was performed in the internal validation data; external incremental value was not evaluated.

The internal calibration output yielded a calibration intercept of approximately 0.00 and a calibration slope of 1.17 for the conventional model, and a calibration intercept of -0.07 and slope of 0.80 for the extended model. The corresponding absolute Brier scores were approximately 0.176 and 0.168, respectively. The paired Brier-score difference was -0.008 (95% CI: -0.013 to -0.003). DCA was evaluated across prespecified threshold probabilities from 0.30 to 0.70. The net benefits for the conventional and extended models were 0.56 and 0.58 at 0.30 (difference 0.02), 0.48 and 0.53 at 0.40 (difference 0.05), 0.37 and 0.48 at 0.50 (difference 0.11), 0.27 and 0.39 at 0.60 (difference 0.12), and 0.14 and 0.25 at 0.70 (difference 0.11). The corresponding treat-all net benefits were 0.563, 0.490, 0.388, 0.235, and -0.020, respectively; treat-none net benefit was 0 at every threshold. Model net-benefit values are reported to two decimal places and treat-all values to three decimal places. These DCA results are interpreted as potential net benefit, not as recommended clinical cutoffs. External incremental value was not evaluated.

Alternative outcome definition and age correlations. Using the alternative screening definition of scores 18–21 for screening-defined probable MCI and scores $\geq$22 for normal cognition, while retaining the exclusion of scores $<$18, 1,364 participants were classified as probable MCI. Under the same stratified 70:30 split, the MLP achieved an internal-validation AUC of 0.804 (95% CI: 0.772–0.834), accuracy of 0.758, sensitivity of 0.780, specificity of 0.692, and F1-score of 0.719. The Brier score was 0.222, the calibration intercept was -0.277, and the calibration slope was 0.552. Education-stratified alternative thresholds were not evaluated. In the training set, Spearman correlations between age and educational attainment, smartphone usage, sex, PSQI, PHQ-9, qi-deficiency constitution, and damp-heat constitution were -0.005, -0.206, 0.050, 0.046, 0.086, 0.053, and 0.034, respectively.

4 SUPPLEMENTARY TABLE S3

Table S3. Characteristics of the primary and external validation samples stratified by screening-defined probable-MCI status. Panel A presents the primary sample.

Variable	Category	Normal cognition (N=1,013)	Probable MCI (N=2,297)	P value
Sex	Male	690 (68.1)	1,304 (56.8)	<0.001
	Female	323 (31.9)	993 (43.2)	
Educational level	Low	482 (47.6)	1,437 (62.6)	<0.001
	Middle	260 (25.7)	494 (21.5)	
	High	271 (26.8)	366 (15.9)	
Living alone	Yes	80 (7.9)	164 (7.1)	0.486
	No	933 (92.1)	2,133 (92.9)	
History of drug and food allergies	Yes	57 (5.6)	181 (7.9)	0.025
	No	956 (94.4)	2,116 (92.1)	
Smoking history	Yes	199 (19.6)	401 (17.5)	0.145
	No	814 (80.4)	1,896 (82.5)	
Alcohol consumption history	Yes	185 (18.3)	370 (16.1)	0.139
	No	828 (81.7)	1,927 (83.9)	
Smartphone usage	Yes	637 (62.9)	1,217 (53.0)	<0.001
	No	376 (37.1)	1,080 (47.0)	
Computer usage	Yes	78 (7.7)	51 (2.2)	<0.001
	No	935 (92.3)	2,246 (97.8)	
Age, years		70.23 ± 7.39	70.72 ± 7.78	0.467
Systolic blood pressure		135.03 ± 17.43	134.36 ± 19.05	0.180
Diastolic blood pressure		79.96 ± 11.16	80.48 ± 11.33	0.195
BMI		23.30 ± 2.98	23.32 ± 3.04	0.703
PSQI		6.40 ± 3.36	6.80 ± 3.65	0.002
PHQ-9		1.92 ± 3.03	2.29 ± 3.34	0.003
MNA-SF		12.18 ± 1.81	12.20 ± 1.90	0.194
Balanced constitution		268 (26.5)	689 (30.0)	0.042
Qi-deficiency constitution		44 (4.3)	128 (5.6)	0.167
Yang-deficiency constitution		102 (10.1)	275 (12.0)	0.126
Yin-deficiency constitution		93 (9.2)	236 (10.3)	0.365
Phlegm-dampness constitution		140 (13.8)	376 (16.4)	0.070
Damp-heat constitution		26 (2.6)	29 (1.3)	0.011
Blood-stasis constitution		22 (2.2)	53 (2.3)	0.909
Qi-stagnation constitution		26 (2.6)	53 (2.3)	0.744

Panel B. External validation sample

Variable	Category	Normal cognition (n=69)	Probable MCI (n=245)
Sex	Male	43 (62.3)	137 (55.9)
	Female	26 (37.7)	108 (44.1)
Educational level	Low	43 (62.3)	175 (71.4)
	Middle	15 (21.7)	47 (19.2)
	High	11 (15.9)	23 (9.4)
Living alone	Yes	10 (14.5)	21 (8.6)
	No	59 (85.5)	224 (91.4)

Variable	Category	Normal cognition (n=69)	Probable MCI (n=245)
History of drug and food allergies	Yes	3 (4.3)	22 (9.0)
	No	66 (95.7)	223 (91.0)
Smoking history	Yes	7 (10.1)	41 (16.7)
	No	62 (89.9)	204 (83.3)
Alcohol consumption history	Yes	12 (17.4)	37 (15.1)
	No	57 (82.6)	208 (84.9)
Smartphone usage	Yes	40 (58.0)	132 (53.9)
	No	29 (42.0)	113 (46.1)
Computer usage	Yes	6 (8.7)	8 (3.3)
	No	63 (91.3)	237 (96.7)
Age, years		70.80 ± 11.47	70.77 ± 7.86
Systolic blood pressure		138.77 ± 20.32	134.96 ± 19.59
Diastolic blood pressure		79.81 ± 10.35	80.80 ± 12.57
BMI		23.14 ± 2.78	23.05 ± 2.92
PSQI		6.26 ± 3.26	7.04 ± 3.80
PHQ-9		1.91 ± 2.69	2.18 ± 3.27
MNA-SF		12.12 ± 2.05	12.12 ± 1.90
Balanced constitution		18 (26.1)	68 (27.8)
Qi-deficiency constitution		1 (1.4)	15 (6.1)
Yang-deficiency constitution		12 (17.4)	29 (11.8)
Yin-deficiency constitution		10 (14.5)	32 (13.1)
Phlegm-dampness constitution		10 (14.5)	36 (14.7)
Damp-heat constitution		0 (0.0)	5 (2.0)
Blood-stasis constitution		1 (1.4)	8 (3.3)
Qi-stagnation constitution		1 (1.4)	4 (1.6)

Values are presented as n (%) or mean ± standard deviation.

5 SUPPLEMENTARY TABLE S4

Table S4. Comparison of the training, internal validation, and external validation samples.

Characteristic	Training set (n=2,317)	Internal validation (n=993)	External validation (n=314)
Screening-defined probable MCI	1,608 (69.4)	689 (69.4)	245 (78.0)
Age, years	70.58 ± 7.44	70.56 ± 8.17	70.78 ± 8.76
Female sex	938 (40.5)	378 (38.1)	134 (42.7)
Low educational level	1,326 (57.2)	593 (59.7)	218 (69.4)
Middle educational level	534 (23.0)	220 (22.2)	62 (19.7)
High educational level	457 (19.7)	180 (18.1)	34 (10.8)
Met possible sarcopenia criteria	2,317 (100)	993 (100)	314 (100)
Grip strength, kg	15.67 ± 6.69	15.50 ± 6.81	15.83 ± 6.98
Smartphone usage	1,303 (56.2)	551 (55.5)	172 (54.8)
PSQI score	6.62 ± 3.57	6.81 ± 3.55	6.87 ± 3.70
PHQ-9 score	2.11 ± 3.13	2.35 ± 3.51	2.12 ± 3.15
Qi-deficiency constitution	117 (5.0)	55 (5.5)	16 (5.1)
Damp-heat constitution	37 (1.6)	18 (1.8)	5 (1.6)

Values are n (%) or mean ± standard deviation. The training and internal-validation columns were generated from the prespecified stratified 70:30 split of the 3,310-participant development cohort. The external sample came from one institution and had a higher probable-MCI proportion and lower proportion with high educational attainment. These distributional differences, together with the smaller external sample, should be considered when interpreting the lower external AUC.

6 SUPPLEMENTARY TABLE S5

Table S5. Variable-level missingness and sequential exclusion flow.

Variable or processing stage	Missing/excluded n	Percentage or remaining n
Variable-level missingness in the post-sarcopenia, pre-final-model dataset (n=6,535)		
Age	0	0.00%
Sex	0	0.00%
Educational attainment	311	4.76%
Living arrangement	0	0.00%
Systolic blood pressure	0	0.00%
Diastolic blood pressure	0	0.00%
BMI	0	0.00%
Drug/food allergy history	1	0.02%
Smoking history	1	0.02%
Alcohol history	1	0.02%
Smartphone use	0	0.00%
Computer use	0	0.00%
PSQI component fields (source-stage)	2,000	30.60%
PHQ-9 score	0	0.00%
MNA-SF score	0	0.00%
Principal TCM constitution category	532	8.14%
Individual CCMQ dimension-score fields	1,402	21.45%
MoCA total score	225	3.44%
Sequential exclusion flow		
Original concatenated source file	–	14,520
Grip-strength data-quality screen	99 excluded	14,421 remaining
Possible-sarcopenia eligibility	7,598 excluded	6,823 remaining
PSQI/MNA-SF component completeness	210 excluded	6,613 remaining
Additional data-quality filters	78 excluded (39+39)	6,535 remaining
Missing MoCA or MoCA <18	2,012 excluded (225+1,787)	4,523 remaining
Complete candidate-predictor/model matrix	1,213 excluded	3,310 remaining

Note: Variable-level counts are reported for processing-stage fields and are not additive because a record may have more than one incomplete field and some questionnaire totals were recalculated from component items. The source-level MoCA accounting is reported separately: 13,773 records had a numeric MoCA total, including 2,649 scores <18, 5,856 scores from 18 to <26 (including two scores of 25.5), and 5,268 scores ≥26. The 225 records without a usable MoCA score in the sequential flow are the residual missing-MoCA records after preceding screening steps, not the complete source-level total of 747.
